# How to report educational videos in robotic surgery: an international multidisciplinary consensus statement

**DOI:** 10.1007/s13304-020-00734-5

**Published:** 2020-03-07

**Authors:** Valerio Celentano, Neil Smart, John McGrath, Ronan A. Cahill, Antonino Spinelli, Ben Challacombe, Igor Belyansky, Hirotoshi Hasegawa, Venkatesh Munikrishnan, Gianluca Pellino, Jamil Ahmed, Filip Muysoms, Avanish Saklani, Jim Khan, Daniel Popowich, Conrad Ballecer, Mark G. Coleman

**Affiliations:** 1 Department of Colorectal Surgery, Queen Alexandra Hospital, Portsmouth Hospitals NHS Trust, Portsmouth, UK; 2 University of Portsmouth, Portsmouth, UK; 3 Exeter Surgical Health Services, Research Unit, Royal Devon & Exeter Hospital, Exeter, Devon UK; 4 Department of Urology, Royal Devon and Exeter NHS Trust, Exeter, UK; 5 University of Exeter Medical School, Exeter, UK; 6 Colorectal Unit, Mater Misericordiae University Hospital, Dublin, Ireland; 7 Section of Surgery and Surgical Specialities, School of Medicine, University College Dublin, Dublin, Ireland; 8 Humanitas Clinical and Research Center – IRCCS, 20089 via Manzoni 56, Rozzano, Milan, Italy; 9 Department of Biomedical Sciences, Humanitas University, Via Rita Levi Montalcini 4, 20090 Pieve Emanuele, Milan, Italy; 10 Department of Urology, Guy’s and St. Thomas’ Hospital NHS Foundation Trust, London, SE1 9RT UK; 11 Department of Surgery, Anne Arundel Medical Center, 2000 Medical Parkway, Suite 100, Annapolis, MD 21401 USA; 12 Department of Surgery, School of Medicine, Keio University, Tokyo, Japan; 13 Institute of Colorectal Surgery, Apollo Hospital, Chennai, India; 14 Department of Medical, Surgical, Neurological, Metabolic, and Ageing Sciences, Universitá Della Campania “Luigi Vanvitelli”, Naples, Italy; 15 Department of Colorectal Surgery, The Royal Bournemouth Hospital NHS Foundation Trust, Bournemouth, UK; 16 Departement of Surgery, Maria Middelares Hospital, Ghent, Belgium; 17 Tata Memorial Hospital, Mumbai, India; 18 Division of Colon and Rectal Surgery, Mount Sinai Hospital, New York, NY USA; 19 Center for Minimally Invasive and Robotic Surgery, Phoenix, AZ USA; 20 University Hospitals Plymouth NHS Trust, Derriford Hospital, Plymouth, UK

**Keywords:** Robotic surgery, Minimally invasive surgery, Surgical videos, Video guidelines, Distance learning, Learning curve

## Abstract

The swift endorsement of the robotic surgical platform indicates that it might prevail as the preferred technique for many complex abdominal and pelvic operations. Nonetheless, use of the surgical robotic system introduces further layers of complexity into the operating theatre necessitating new training models. Instructive videos with relevant exposition could be optimal for early training in robotic surgery and the aim of this study was to develop consensus guidelines on how to report a robotic surgery video for educational purposes to achieve high quality educational video outputs that could enhance surgical training. A steering group prepared a Delphi survey of 46 statements, which was distributed and voted on utilising an electronic survey tool. The selection of committee members was designed to include representative surgical trainers worldwide across different specialties, including lower and upper gastrointestinal surgery, general surgery, gynaecology and urology. 36 consensus statements were approved and classified in seven categories: author’s information and video introduction, case presentation, demonstration of the surgical procedure, outcomes of the procedure, associated educational content, review of surgical videos quality and use of surgical videos in educational curricula. Consensus guidelines on how to report robotic surgery videos for educational purposes have been elaborated utilising Delphi methodology. We recommend that adherence to the guidelines presented could support advancing the educational quality of video outputs when designed for training.

## Introduction

Robotic assisted surgery has the potential to surmount some of the restraints of laparoscopy, presenting an immersive 3-dimensional depth of field, articulating instruments and a stable camera platform [1]. The swift endorsement of the robotic surgical platform indicates that it might prevail as the preferred technique for many complex abdominal and pelvic operations. Nonetheless, use of the surgical robotic system introduces further layers of complexity into the operating theatre, including a change in the conventional surgeon and trainee relationship, new highly developed technology, different motor and visual skills, and challenges in communication, thus necessitating new training models [2]. The training in new procedures including robotic surgery is characterised by changes in practice over time, or the proficiency curve [3], which has been recognised as one of the main barriers for surgeons to embrace robotic surgery, alongside costs and lack of drive from the hospitals [4].

Each robotic system is costly and is likely to be highly demanded for clinical use; as a consequence simulation training exercises may need to happen outside of clinical work time to access this resource, which can inhibit its use. Further challenges, especially for trainees, comprehend the competition for trainee time for other highly set educational activities, clinical commitments and working hours restrictions [5]. Observing live operating, attending educational workshops and seminars are all valuable resources but necessitate surgeons to interrupt their clinical activity to attend dedicated training sessions [6]. Instructive videos with relevant exposition could be exemplary for early training in robotic surgery [7] and can be developed even with basic prior video editing background [8]. The video output has the convenience that explicatory operations are chosen in advance and the educational content can be outlined beforehand [9]. Surgical trainers acknowledge online videos as a valuable teaching aid [10] that maximizes trainees’ learning and skill improvement in view of the backdrops of time constraints and productivity requirements [11], but the reliability of a significant part of highly viewed freely available content continues to remain debatable, as not all video outputs are trustworthy and some may not demonstrate procedures based on strong evidence [12].

On the basis of these premises, the aim of this study was to develop consensus guidelines on how to report a robotic surgery video for educational purposes to achieve high quality educational video outputs that could enhance surgical training.

## Methods

The guidelines were established according to The Appraisal of Guidelines Research and Evaluation Instrument II (Agree II, https://www.agreetrust.org/agree-ii). A steering committee was selected to incorporate surgical trainers as contributors across several specialties such as general surgery, gynaecology, urology and lower and upper gastrointestinal surgery. Committee members were selected on the basis of previously published experience in guidelines development [13] on distance learning in surgery [12], minimally invasive surgery training programme development [14] and dissemination of online surgical videos [15]. 18 experts made up this committee.

A steering subcommittee comprising 10 members from 5 countries and 4 surgical specialties defined the consensus report. The steering committee was accountable for the selection of the survey items and statements were agreed upon following teleconferences, e-mails and face-to-face meetings. An electronic survey tool (Enalyzer, Denmark, www.enalyzer.com) was used for the voting round of the Delphi survey items after 46 statements were prepared by the consensus committee.

The Delphi methodology is a generally adopted procedure with a systematic progression of repeated rounds of voting for attaining agreement among a board of experts [16]. The experts vote anonymously to a minimum two rounds survey; participants expressing a vote against a survey item need to complete a reviewed statement with an explanation for their choice [17]. During the last round of the survey, participants do not have any more the opportunity to amend the items, and therefore only a binary accept or reject option is available. The required threshold for acceptance of a survey item into the consensus statements was of ≥ 80% [18, 19]. Feedbacks on the items not reaching 80% agreement were revised by the consensus guidelines members after the first round and statements were amended and submitted again for a second round of the survey. The finalised consensus guidelines were disseminated together with the draft article to all members of the committee.

## Results

All 18 representatives of the consensus committee answered both the first and the second round of the Delphi survey. The first Delphi analysis comprised 46 items. The statements not achieving the minimum required 80% agreement at the first round were reviewed and circulated for a second vote. 36 consensus statements were finally agreed and are summarised in seven categories with the rate of agreement shown in Table 1. Rejected survey items are presented in Table 2.

**Table 1:** 36 consensus statements approved by the committee, with rate of agreement

	Authors information and video introduction	% Agreement
1	The video must include authors’ information such as names, Institution(s), country, year of surgery. Contact details of the corresponding author must be provided	83.3
2	The title of the video must include the name of the procedure performed and of the pathology treated. ‘Robotic assisted’ or ‘hybrid robotic/laparoscopic assisted’ should be specified in the title	100
3	If the video is intended for training this should be specified and specific learning objectives could be presented. Aim of the video and relevance of the case presented should be stated	88.8
4	It is desirable to describe the experience of the surgeon or institution in performing the procedure	80
5	Patient consent should be obtained	86.6
6	A conflict of interest disclosure must be present	92
Case presentation		
7	All radiology images, videos and reports should be anonymised and the name of the patient and confidential data should never be mentioned. All patient recognisable body parts such as eyes and tattoos should be obscured	100
8	The video should include one or more slides or audio-commentary with formal presentation of the case, including age, sex, American society of Anaesthesiologist score (ASA), body mass index (BMI), indication for surgery, comorbidities and history of previous surgery. Preoperative staging and neoadjuvant treatment should be detailed in case of malignancy	86.6
9	Results of preoperative imaging should be presented	100
Demonstration of the surgical procedure		
10	The name of the robotic system used must be detailed including device version specification	93.3
11	The position of the patient on the operating table must be demonstrated or illustrated schematically through a diagram, including variations during the surgery	94.4
12	Docking should be clearly explained and schematically represented if not possible to have an operating room picture	86.6
13	Double docking, hybrid or single docking should be explained and docking time detailed	86.6
14	The instruments and trocars controlled by the first assistant should be detailed	83.3
15	Type of robotic instruments used should be detailed specifying in which robotic arm	86.6
16	The position of the robotic and of the assistant’s trocars must be detailed	100
17	The site for specimen extraction should be demonstrated or mentioned	100
18	Relevant additional intraoperative investigations should be mentioned or demonstrated	96.4
19	Details of special equipment needed for the procedure should be provided, such as vessel sealer devices, wound protectors, manipulators and surgical staplers	100
20	The surgical procedure should be presented in a standardised step by step “modular” fashion	93.3
21	Every chapter should be clearly introduced and explained. The intraoperative findings need to be demonstrated, with constant reference to anatomical landmarks and surgical planes with the aid of telestration if available	93.3
22	Additional manoeuvers and suggestions to face “progression failure” should be demonstrated—for instance additional ports or assistants, change of the position of the patient or rescue manoeuvres in case of unexpected events such surgical stapler malfunction or equipment failure	82.3
23	Describing the criteria for conversion to laparoscopic/open surgery and the site of the incision in case of conversion might be useful in training videos	93.7
24	If a hybrid laparoscopic/robotic procedure is performed, the laparoscopic steps should be mentioned in a training video	86.6
Outcomes of the procedure		
25	Outcomes of the procedure must be presented, including total procedure time, operating time, blood loss, length of hospital stay and postoperative morbidity	87.5
26	Histopathology assessment of the specimen should be presented. In case of malignancy number of retrieved lymph nodes and TNM staging should be detailed. Pictures of the specimen are desirable	81.2
Associated educational content		
27	Additional educational content must be included. Telestration, diagrams, photos, snapshots and tables should be used to demonstrate anatomical landmarks, relevant or unexpected findings	83.3
28	An accessory slide with description of pitfalls and errors and how to avoid mistakes it is desirable in training videos	93.3
29	Audio/written commentary in English language must be provided	88.9
Review of surgical videos quality		
30	Image quality should be assessed. When excessive smoke, low definition or suboptimal views are present for more than 25% of the duration of the procedure, the video should be rejected for poor image quality	94.4
31	Robotic videos are most efficient at 1.3–1.5 speed. Video speed should be indicated in the respective video segments (e.g., 2 ×, 4 ×, 0.5 ×)	94.1
Use of surgical videos in educational curricula		
32	Routine video-recording of the procedure and review with feedback sessions should be mandatory in every training program	83.3
33	Video recording can be useful for continue professional development even at the completion of the learning curve, to review unusual findings and to reflect on complications and outcomes	94.4
34	Videos demonstrating unusual cases and management of intraoperative complications should be shared at conferences	100
35	Formative assessment of the surgical performance should involve peer-review of unedited videos, using standardised assessment tools	84.6
36	The web platform should record the number of times the video has been watched for audit purposes. Moreover, it should allow comments and webchats to facilitate feedback and interaction amongst trainers and trainees	88.9

**Table 2:** Statements that did not reach consensus agreement

	Rejected statements	% Agreement
1	It should be specified if the video was presented at national/international meetings or recorded during a live broadcast	60
2	Theatre layout, the position of the surgical and anaesthetic team should be demonstrated, including scrub nurse position and position of extra assistants	50
3	Educational videos must undergo formal peer review prior to publication. It should be stated if the video has been peer reviewed prior to publication	73.3
4	Peer review should assess not only the safety of the procedure performed, but also the supplementary educational content presented	73.3
5	Peer review should be undertaken by both surgical trainers and trainees	40
6	Videos should be amended and resubmitted, where possible, according to the reviewers’ comments with a point by point answer	66.6
7	An unedited copy of the video should be made available for review either on request from the author or via the publisher	73.3
8	Follow-up duration, and follow-up pathways should be detailed	32
9	A comparison with other studies should be presented as an accessory slide	47
10	Essential references should be provided	60

## Discussion

Before undertaking robotic surgery clinical training operating on real patients with expert supervision, novice surgeons must first become familiar with the robotic interface [20] by attending dedicated courses and using online educational material and simulators according to a structured approach. Intraoperative mentorship and structured feedback from colleagues are beneficial even beyond completion of residency training, but time constraints and hierarchy can limit significantly implementation [21]. Surgeon video review leads to improved techniques and outcomes [22] with postoperative video debriefing being shown as an effective educational tool leading to reduced adverse events [23]. Video based peer feedback through social networking allows surgeons to receive mentorship at a convenient time beyond geographical limitations [24] and holds promises to become an essential part of continual professional development, provided patient privacy and consenting is maintained. E-modules and video training are extremely valuable educational methods, however their use its not exclusive and only effective if integrated within a structured training program, including simulation training, dry and wet lab activity. Moreover, proctoring plays an essential role in guaranteeing patients’ safety when operations are performed during the initial part of the surgeons’ learning and proficiency curve.

One of the main strengths of our research is that we have collated the expertise of several international committee members across different surgical specialties to establish consensus agreement on how to present a robotic surgery video developed for the scope of surgical education, with the main aim to enhance the educational content of videos by introducing a reference standard to reduce the variability in the quality, trustworthiness and educational accuracy of online robotic surgery videos, as we previously published in laparoscopic surgery [13]. Consensus guidelines, generally reported as a checklist, flow diagram, or explicit text, clarify a required set of information for a complete and clear account for reporting what was done and found during a study, highlighting factors potentially accountable for bias introduction into the project [25]. We acknowledge the lack of previously published guidelines for reporting of a robotic surgery video and, as such, the quality of these video outputs is very heterogeneous. To enhance the educational quality of published robotic surgery videos, especially when intended for training, the logical progression is to set a reference standard by introducing consensus agreement. Technical competence is a prerequisite for independent practising and encompasses understanding of pertinent anatomy, natural evolution of the disease, indications, steps and possible complications of the surgical technique [26] which are the reasons why additional educational content should be included in training videos.

Procedural competency can depend on the number of cases performed under supervision [27], which is consistent with the theory of deliberate practice, implying that proficiency is not only associated with the volume of cases but also with the time used practising with constructive feedback [28]. As a consequence, objective assessments must be applied to evaluate procedural competence focusing on the safety of the performance rather than the number of cases completed and distance learning in surgery should not only be confined to observing a video of another surgeon operating, but also incorporate examining the trainees’ own performance, by revising the video with peers and trainers. It has been demonstrated that constructive feedback can enhance performance [29], and therefore it must be an essential component of training in robotic surgery, in spite of representing a shift from the more classic methods of surgical training [30]. Commensurate training for new technologies is essential for the safe introduction into the wider surgical community. Credentialing aims to assure safety for patients, and gives confidence to hospitals that adequate training has been achieved, which is the reason why it is a requirement in many institutions [31]. Peer review of surgical videos submitted according to standardised criteria, could provide an effective tool for maintaining credentialing for robotic surgeons.

The proficiency curve in robotic surgery concerns the whole team [32], not just the surgeon, and we must acknowledge this as a limitation of these guidelines, which may provide limited benefit to the anaesthetists, nursing staff and operating department practitioners, who all require training as part of the robotic surgery team [33]. Teamwork and communication are paramount for safe and effective performance, particularly in robotic surgery [34] which introduced physical distance between the surgical team members and the patient providing changes to the spatial configuration of the operating room [35]. How the lack of face to face interaction can affect team communication has not been explored in these guidelines, which focus on surgeon’s technical skills [36]. Surgical trainees acknowledge highly informative videos reporting patients’ data and procedure outcomes, and integrated with supplementary educational material such as screenshots and diagrams to help identification of anatomical structures [37]. We must recognise another limitation of these guidelines is the time needed for producing such high-quality video outputs, with several gigabytes required for storage and sharing of high definition robotic videos that can be produced and uploaded with minimal technical skills [38]. It is important to acknowledge that there is minimal data available in the published literature to base this consensus statement on high quality evidence, which may explain why almost none of the accepted statements reached 100% agreement. This lack of endorsement for some statements is not uncommon when using Delphi methodology, however, a threshold for approval of 80% was selected and transparency was ensured by publishing both the accepted and rejected statements with correspondent rate of agreement. Nevertheless, the Delphi process with pre-set objectives is an accepted methodology to reduce the risk of individual opinions prevailing and the invited co-authors of these practice guidelines have previously reported on the topic of surgical videos availability [39], quality [12], content standardisation [13] and use by surgeons in training [37].

There is currently no standard accreditation or regulation for medical videos as training tools [40]. The HONCode [41] is a code of conduct for online medical and health platforms, but this applies to all web content and is not specific for audio-visual material. We propose that following these guidelines could help improve video quality and offer a standardised tool for use in quality evaluation of video materials presented for publication or conferences, although we appreciate that they were not developed with this purpose and further validation research would be needed.

## Conclusions

Consensus guidelines on how to report robotic surgery videos for educational purposes have been developed utilising Delphi methodology. Adherence to the presented guidelines could help enhance the educational value of video outputs when used for the scope of surgical training.

## References

[1] Jayne D, Pigazzi A, Marshall H (2017). Effect of robotic-assisted vs conventional laparoscopic surgery on risk of conversion to open laparotomy among patients undergoing resection for rectal cancer: the ROLARR randomized clinical trial. JAMA.

[2] Zorn KC, Gautam G, Shalhav AL (2009). Training, credentialing, proctoring and medicolegal risks of robotic urological surgery: recommendations of the Society of urologic robotic surgeons. J Urol.

[3] Harrysson IJ, Cook J, Sirimanna P (2014). Systematic review of learning curves for minimally invasive abdominal surgery. Ann Surg.

[4] Benmessaoud C, Kharrazi H, MacDorman KF (2011). Facilitators and barriers to adopting robotic-assisted surgery: contextualizing the unified theory of acceptance and use of technology. PLoS ONE.

[5] Tarr ME, Rivard C, Petzel AE (2014). Robotic objective structured assessment of technical skills: a randomized multicenter dry laboratory training pilot study. Female Pelvic Med Reconstr Surg.

[6] Schurr M, Arezzo A, Buess G (1999). Robotics and systems technology for advanced endoscopic procedures: experiences in general surgery. Eur J Cardiothorac Surg.

[7] Hall JC (2002). Imagery practice and the development of surgical skills. Am J Surg.

[8] Tolerton SK, Hugh TJ, Cosman PH (2012). The production of audiovisual teaching tools in minimally invasive surgery. J Surg Educ.

[9] Rocco B, Lorusso A, Coelho RF, Palmer KJ, Patel VR (2009). Building a robotic program. Scand J Surg.

[10] Abdelsattar JM, Pandian TK, Finnesgard EJ (2015). Do you see what I see? How we use video as an adjunct to general surgery resident education. J Surg Educ.

[11] Gorin MA, Kava BR, Leveillee RJ (2011). Video demonstrations as an intraoperative teaching aid for surgical assistants. Eur Urol.

[12] Celentano V, Browning M, Hitchins C (2017). Training value of laparoscopic colorectal videos on the world wide web: a pilot study on the educational quality of laparoscopic right hemicolectomy videos. Surg Endosc.

[13] Celentano V, Smart N, Cahill R (2018). LAP-VEGaS practice guidelines for reporting of educational videos in laparoscopic surgery: a joint trainers and trainees consensus statement. Ann Surg.

[14] Coleman M, Rockall T (2013). Teaching of laparoscopic surgery colorectal. The Lapco model. Cir Esp.

[15] Jacob BP (2012) International hernia collaboration. Facebook. www.facebook.com/groups/herniacollab/. Accessed 25 Nov 2019

[16] Linstone HA, Turoff M (1975). The Delphi method techniques and applications.

[17] Varela-Ruiz M, Díaz-Bravo L, García-Durán R (2012). Description and uses of the Delphi method for research in the healthcare area. Inv Ed Med.

[18] Boulkedid R, Abdoul H, Loustau M (2011). Using and reporting the Delphi method for selecting healthcare quality indicators: a systematic review. PLoS ONE.

[19] Lynn MR (1986). Determination and quantification of content validity. Nurs Res.

[20] Schreuder HW, Wolswijk R, Zweemer RP, Schijven MP, Verheijen RH (2012). Training and learning robotic surgery, time for a more structured approach: a systematic review. BJOG.

[21] Hu YY, Peyre SE, Arriaga AF (2012). Postgame analysis: using video-based coaching for continuous professional development. J Am Coll Surg.

[22] Walsh PC, Marschke P, Ricker D (2000). Use of intraoperative video documentation to improve sexual function after radical retropubic prostatectomy. Urology.

[23] Hamad GG, Brown MT, Clavijo-Alvarez JA (2007). Postoperative video debriefing reduces technical errors in laparoscopic surgery. Am J Surg.

[24] Carter SC, Chiang A, Shah G (2015). Video-based peer feedback through social networking for robotic surgery simulation: a multicenter randomized controlled trial. Ann Surg.

[25] Larson EL, Cortazal M (2012). Publication guidelines need widespread adoption. J Clin Epidemiol.

[26] Sood A, Jeong W, Ahlawat R (2015). Robotic surgical skill acquisition: what one needs to know?. J Minim Access Surg.

[27] Mackenzie H, Ni M, Miskovic D (2015). Clinical validity of consultant technical skills assessment in the English national training programme for laparoscopic colorectal surgery. Br J Surg.

[28] Ericsson KA (2004). Deliberate practice and the acquisition and maintenance of expert performance in medicine and related domains. Acad Med.

[29] Nisar PJ, Scott HJ (2001). Key attributes of a modern surgical trainer: perspectives from consultants and trainees in the United Kingdom. J Surg Educ.

[30] Rolfe I, McPherson J (1995). Formative assessment: how am I doing?. Lancet.

[31] Bell S, Carne P, Chin M, Farmer C (2015). Establishing a robotic colorectal surgery programme. ANZ J Surg.

[32] Jayaraman S, Davies W, Schlachta C (2009). Getting started with robotics in general surgery with cholecystectomy: the Canadian experience. Can J Surg.

[33] Herron D, Marohn M (2008). Group TS-MRSC: a consensus document on robotic surgery. Surg Endosc.

[34] Randell R, Greenhalgh J, Hindmarsh J (2014). Integration of robotic surgery into routine practice and impacts on communication, collaboration, and decision making: a realist process evaluation protocol. Implement Sci.

[35] Lai F, Entin E (2005). Robotic surgery and the operating room team. Proc Hum Factors Ergon Soc Annu Meet.

[36] Sgarbura O, Vasilescu C (2010). The decisive role of the patient-side surgeon in robotic surgery. Surg Endosc.

[37] Celentano V, Smart N, Cahill RA, McGrath JS, Gupta S, Griffith JP, Acheson AG, Cecil TD, Coleman MG (2018). Use of laparoscopic videos amongst surgical trainees in the United Kingdom. Surgeon.

[38] Dinscore A, Andres A (2010). Surgical videos online: a survey of prominent sources and future trends. Med Ref Serv Q.

[39] Celentano V, Pellino G, Coleman MG (2019). Lack of online video educational resources for open colorectal surgery training. ANZ J Surg.

[40] Langerman A, Grantcharov TP (2017). Are we ready for our close-up?: why and how we must embrace video in the OR. Ann Surg.

[41] Health on the net foundation. The HON Code of Conduct for medical and health Web sites (HONcode). https://www.healthonnet.org/. Accessed 10 Dec 2019

